# Correction of NR2E3 Associated Enhanced S-cone Syndrome Patient-specific iPSCs using CRISPR-Cas9

**DOI:** 10.3390/genes10040278

**Published:** 2019-04-05

**Authors:** Laura R. Bohrer, Luke A. Wiley, Erin R. Burnight, Jessica A. Cooke, Joseph C. Giacalone, Kristin R. Anfinson, Jeaneen L. Andorf, Robert F. Mullins, Edwin M. Stone, Budd A. Tucker

**Affiliations:** Institute for Vision Research, Department of Ophthalmology and Visual Sciences, University of Iowa, Iowa City, IA 52241, USA; laura-bohrer@uiowa.edu (L.R.B.); luke-wiley@uiowa.edu (L.A.W.); erin-burnight@uiowa.edu (E.R.B.); jessica-cooke@uiowa.edu (J.A.C.); joseph-giacalone@uiowa.edu (J.C.G.); kristin-anfinson@uiowa.edu (K.R.A.); jeaneen-andorf@uiowa.edu (J.L.A.); robert-mullins@uiowa.edu (R.F.M.); edwin-stone@uiowa.edu (E.M.S.)

**Keywords:** induced pluripotent stem cell (iPSC), clustered regularly interspaced short palindromic repeats (CRISPR), homology-directed repair (HDR), Enhanced S-Cone Syndrome (ESCS), *NR2E3*

## Abstract

Enhanced S-cone syndrome (ESCS) is caused by recessive mutations in the photoreceptor cell transcription factor *NR2E3*. Loss of *NR2E3* is characterized by repression of rod photoreceptor cell gene expression, over-expansion of the S-cone photoreceptor cell population, and varying degrees of M- and L-cone photoreceptor cell development. In this study, we developed a CRISPR-based homology-directed repair strategy and corrected two different disease-causing *NR2E3* mutations in patient-derived induced pluripotent stem cells (iPSCs) generated from two affected individuals. In addition, one patient’s iPSCs were differentiated into retinal cells and *NR2E3* transcription was evaluated in CRISPR corrected and uncorrected clones. The patient’s c.119-2A>C mutation caused the inclusion of a portion of intron 1, the creation of a frame shift, and generation of a premature stop codon. In summary, we used a single set of CRISPR reagents to correct different mutations in iPSCs generated from two individuals with ESCS. In doing so we demonstrate the advantage of using retinal cells derived from affected patients over artificial in vitro model systems when attempting to demonstrate pathophysiologic mechanisms of specific mutations.

## 1. Introduction

Enhanced S-cone syndrome (ESCS) is an autosomal recessive retinopathy that results from mutations in the photoreceptor cell transcription factor, *NR2E3* (Nuclear Receptor Subfamily 2, Group E, Member 3). *NR2E3*, which is specifically expressed in the outer nuclear layer of the human retina [1], is a direct transcriptional target of NRL, a key regulator of photoreceptor cell genesis. Work in animal models has shown that *NR2E3* acts to repress cone photoreceptor cell gene expression and promote rod photoreceptor cell fate commitment. Specifically, loss of *NR2E3* function hinders rod photoreceptor cell development and drives over-expansion of the S-opsin-positive cone photoreceptor cell population (i.e., blue cones) [2,3,4,5,6], which is normally the least prevalent of the photoreceptor cell subtypes.

Patients with ESCS present with increased sensitivity to blue light (due to overabundance of short wavelength sensitive S-cones), early onset impairment of night vision (due primarily to lack of functional rod development) and varying degrees of sensitivity to green and red light (due to varying abundance of medium wavelength sensitive M-cones and long wavelength sensitive L-cones). Although the disease is progressive in nature, clinically evident retinal degeneration is often highly-variable, ranging from a relatively mild and nearly static disorder in some individuals to a very severe and progressive disease in others [1,7,8,9,10].

To date, more than 33 different disease-causing mutations in *NR2E3* have been described [1,11,12]. In addition to ESCS, mutations in NR2E3 can also cause more severe forms of retinal disease, including Goldmann-Farve syndrome (GFS) or autosomal dominant retinitis pigmentosa (adRP). Of these mutations, the majority are in either DNA- (e.g., c.219G>C; p.(Arg73Ser)) or ligand-binding (e.g., c.932G>A; p.(Arg311Gln)) domains of the NR2E3 protein [11,13]. That said, one of the most common *NR2E3* mutations reported in the United States is c.119-2A>C [13], which falls within the canonical splice acceptor site of intron 1. To demonstrate the functional effect of this mutation, Bernal and colleagues transiently transfected COS7 cells with an expression plasmid containing a copy of the *NR2E3* gene harboring the c.119-2A>C variant [14]. Sequence analysis revealed that in addition to the normal transcript, an aberrant transcript that lacked exon 2 and contained a premature stop codon in exon 3 was also present [14].

Understanding how different disease-causing *NR2E3* genotypes alter retinal development and give rise to the observed spectrum of clinical outcomes is of particular interest and relevance to investigators attempting to develop treatments based on photoreceptor cell replacement. Specifically, by understanding exactly how genes such as *NR2E3* function, we may be able to strategically guide developing photoreceptor cells down a more cone-selective path (i.e., alter *NR2E3* function to increase cone genesis without causing widespread photoreceptor cell degeneration). To evaluate the effects of different *NR2E3* variants on photoreceptor cell fate, we have used CRISPR-based genome editing of human patient-derived induced pluripotent stem cells (iPSC). Human iPSCs have several advantages over animal models for this type of work. Unlike animal models, iPSCs can be generated from patients with known disease-causing variants on genetic backgrounds that are demonstrably permissive of the disease. By correcting the patients’ mutations one at a time, one can readily evaluate the role of a specific variant on gene expression and phenotypic outcome. Correlating these in vitro findings with the patients’ clinical history may provide a better understanding of how variants in each of the different *NR2E3* domains influence photoreceptor cell fate decisions.

To correct retinal disease-causing variants in patient-derived iPSCs, we and others have used CRISPR-based genome editing [15,16,17,18,19,20,21,22]. Unlike the more cumbersome ZFN- and TALEN-based approaches, which require the development of elaborate genomic targeting complexes, the CRISPR method relies on the use of small single guide RNAs (sgRNAs) that can be easily synthesized to direct human codon-optimized Cas9 nuclease to specific genomic targets. Cas9 induces double-strand DNA breaks that can subsequently be repaired via fairly error-prone non-homologous end joining (NHEJ), or via the more precise homology-directed repair (HDR) mechanism [15]. The advantage of the HDR-based strategy, even for mutations within the deep intronic space where NHEJ could be used, is that a single HDR repair template and set of sgRNAs is often sufficient to correct a variety of different mutations that are contained within a limited genomic space (e.g., an HDR template spanning exons 1 through 3 would cover both the above-mentioned c.119-2A>C splice site mutation and the p.(Arg73Ser) variant contained within the *NR2E3*-DNA binding element). By using the same set of reagents to correct a variety of mutations in different cell lines, one can control for differences related to reagent variability while increasing throughput.

In this study, we developed a CRISPR-Cas9-based HDR strategy to correct two different *NR2E3* mutations in iPSCs generated from two patients with clinically-diagnosed and molecularly-confirmed ESCS: Patient 1 harbors homozygous c.119-2A>C mutations, and Patient 2 harbors compound heterozygous p.(Arg73Ser) and p.(Arg311Gln) mutations. The close proximity of c.119-2A>C and p.(Arg73Ser) allowed the same sgRNA and HDR reagents to be used to correct iPSC lines generated from both individuals. Genomic correction of both mutations was confirmed by PCR and Sanger sequencing. To demonstrate the utility of this system for elucidating disease mechanism, iPSCs obtained from Patient 1, both before and after CRISPR correction, were differentiated down a photoreceptor cell lineage. Analysis of the *NR2E3* transcript revealed that, prior to CRISPR correction, patient-derived retinal cells completely lack wild-type messages and instead express a mutant transcript that contains a portion of intron 1, which causes a frameshift and the creation of a premature stop codon. Following monoallelic CRISPR correction, the expression of the wild-type *NR2E3* transcript was restored.

## 2. Materials and Methods

### 2.1. Patient-Derived iPSCs

This study was approved by the Institutional Review Board of the University of Iowa (project approval #200202022) and adhered to the tenets set forth in the Declaration of Helsinki. The dermal fibroblast-derived iPSCs used in this study were generated from two patients with molecularly confirmed ESCS. These previously described cell lines were generated using current good manufacturing practices in a clean room environment and validated using scorecard and karyotypic analysis [23]. Consistent with the patients’ clinical diagnoses and unlike control individuals, differentiation of these lines gave rise to S-opsin-positive blue cone photoreceptor cell-dominated retinal organoids, which lack rod photoreceptor cells [24].

### 2.2. Cloning of CRISPR-Cas9 and HDR Donor Constructs

The sgRNAs used in this study were designed to target the region of *NR2E3* that contains the c.119-2A>C (Patient 1) and c.219G>C; p.(Arg73Ser) (Patient 2) mutations using the Benchling platform (www.benchling.com) or the Optimized CRISPR Design Tool (crispr.mit.edu). Guides were cloned into our previously described bicistronic construct expressing a human codon-optimized *Streptococcus pyogenes Cas9* (*spCas9*) nuclease [17,18]. A homology-directed repair (HDR) construct was synthesized by GenScript. As described previously, the HDR plasmid contains 450–750 bp of homologous sequence flanking a floxed puromycin resistance gene followed by the Herpes Simplex Virus type 1 thymidine kinase (vTK) gene [16].

### 2.3. Screening of NR2E3-specific sgRNAs

sgRNA and CRISPR-Cas9 constructs were transfected into HEK293T cells (ATCC) using Lipofectamine 2000 (Thermo Fisher Scientific) and cleavage was assessed via a T7 endonuclease 1 (T7E1) assay using *NR2E3*-specific primers (Table S1) as described previously [17,18]. Control iPSCs were transfected with 2 μg of plasmid using the Neon transfection system as described previously [16].

### 2.4. Delivery of sgRNA-Cas9 and the HDR Contruct to NR2E3-Patient-Specific iPSCs

The CRISPR-Cas9 machinery was delivered to iPSCs and analyzed as described previously [16,17,18]. Briefly, the sg4-spCas9 and HDR plasmids were transfected at a 1:2 molar ratio into patient iPSCs with Lipofectamine Stem (Thermo Fisher Scientific; Patient 1) or Neon electroporation (Thermo Fisher Scientific; Patient 2). Puromycin (0.5 μg/mL) selection was performed as previously described [16], and surviving colonies were PCR screened for HDR incorporation and sequenced to confirm correction. The puroR-vTK cassette was removed by Lipofectamine Stem transfection of Cre recombinase, and cells were treated with 40–400 nM Ganciclovir to select for those with vTK removal. The top off-target sites (Table S2) were determined using the Benchling platform (https://benchling.com/) and analyzed using the T7E1 assays as described above.

### 2.5. Retinal Differentiation

Undifferentiated iPSCs cultured on Laminin-521 coated culture dishes in Essential 8 medium (Thermo Fisher Scientific) were transitioned to Matrigel (Corning) coated culture dishes in mTESR1 medium (StemCell Technologies). Transitioned cells were passaged every 4–5 days using 1mg/ml dispase (Thermo Fisher Scientific). Differentiation was initiated through embryoid body (EB) formation as previously described [25]. Briefly, EBs were generated on ultra-low adhesion plates and transitioned from mTESR1 to neural induction medium (NIM - DMEM/F12 (1:1), 1% N2 supplement, 1% non-essential amino acids, 1% Glutamax (Thermo Fisher Scientific), 2 μg/ml heparin (Sigma) and 0.2% Primocin (Invivogen)) over a three day time period. EBs were maintained free-floating in NIM until day 7, at which time they were induced to adhere to tissue culture-treated plates overnight in NIM supplemented with 25% fetal bovine serum (FBS). Media was replaced with NIM minus FBS on day 8 and the adherent EBs were fed with NIM every other day until day 16. On day 16, the entire EB outgrowth was mechanically lifted using a cell scraper and transferred to retinal differentiation medium (RDM - DMEM/F12 (3:1), 2% B27 supplement, 1% non-essential amino acids, 1% Glutamax and 0.2% Primocin). Three-dimensional aggregates maintained in RDM give rise to both retinal organoids and non-retinal neurospheres that can be isolated based on their morphological appearances. Retinal organoids were isolated and dissociated at 5–6 weeks using Accutase (StemCell Technologies) and cells were subsequently plated onto laminin (Sigma) coated tissue culture plates. Cells were maintained in RDM and collected at a series of time points for RNA analysis.

### 2.6. Reverse Transcription PCR

Total RNA was extracted from cells using either the NucleoSpin RNA extraction kit (Machery-Nagel) or TRIzol (Thermo Fisher Scientific) following the manufacturer’s protocol. cDNA was generated with the High Capacity cDNA Reverse Transcription Kit (Applied Biosystems) using 200 ng of RNA template. *NR2E3* transcript was amplified using BIOLASE DNA polymerase (Bioline) and *NR2E3* specific primers (Table S1). PCR products were separated on an agarose gel and bands were excised, purified using the QIAquick Gel Extraction Kit (Qiagen) and subcloned using the PCR2.1 TOPO TA cloning kit (Invitrogen). Colonies were subsequently picked and Sanger sequenced using M13(-20)F or M13R primers.

## 3. Results

### 3.1. Testing CRISPR-Cas9 Guide Cleavage in HEK293T Cells and iPSCs

The goal of this study was to develop a CRISPR-Cas9 homology-directed repair strategy suitable for the correction of disease-causing mutations in iPSCs generated from two independent patients with molecularly confirmed *NR2E3-*associated enhanced S-cone syndrome (Figure 1A,B). As enhanced S-cone syndrome has a recessive mode of inheritance (such that correction of only one allele would be expected to mitigate the disease phenotype) and the p.(Arg73Ser) mutation on the paternal allele of Patient 2 is within 100 bps of the c.119-2A>C mutations in Patient 1, we hypothesized that a single CRISPR-Cas9 HDR cassette would be sufficient for the correction of iPSCs generated from both individuals. We began by designing and testing 5 different sgRNAs that utilized the spCas9 PAM sites. To determine the efficiency of cleavage, we evaluated nonhomologous end joining (NHEJ) using T7E1 assays in HEK293T cells transfected with each sgRNA and spCas9. While no cleavage was detected in controls (i.e., untransfected cells), all 5 guides induced cleavage and indel formation with similar efficiencies in the transfected cells (Figure 1C). The ability of all 5 guides to direct specific DNA cleavage was subsequently evaluated in iPSCs generated from a normal non-diseased individual. As with HEK293T cells, all 5 guides induced DNA cleavage and indel formation; however, more robust cleavage was detected in cells that received sg2 and sg4 (Figure 1D). As the PAM sequence used by sg4 could be readily modified with a synonymous mutation in the HDR cassette that would prevent re-cleavage events without altering the predicted amino acid sequence, this guide was chosen for all subsequent patient-specific genome repair experiments.

### 3.2. CRISPR Correction of the NR2E3 c.119-2A>C mutation in Patient-Specific iPSCs using Homology-Directed Repair (HDR)

To correct the c.119-2A>C and p.(Arg73Ser) variants in iPSCs generated from the patients described in Figure 1, we designed an HDR donor cassette with ~500 bps of homologous sequence upstream and downstream of the sg4 cleavage site (Figure 2A). As indicated above, to prevent re-cleavage events, we included a synonymous variant in the sg4 PAM site (CGG>CGA). To select for cells that incorporated the HDR sequence, a puromycin resistance cassette under the control of the mPGK promoter was added to intron 1 as previously described [16,18]. In addition, the stop codon in the puromycin resistance sequence was replaced with a porcine 2A peptide (P2A) coding sequence, followed by the Herpes Simplex Virus type 1 thymidine kinase (vTK) gene (thymidine kinase phosphorylates ganciclovir, a nucleoside analog, which disrupts DNA synthesis and induces cell death) and a downstream polyadenylation sequence (PA) [16]. The entire cassette, mPGK-PuroR-vTK-PA, was flanked by loxP sites that enabled removal of the cassette via Cre recombinase [16]. Following Cre recombinase transfection, treatment of cells with ganciclovir allowed for the selection of cells that had lost the cassette [16]. Patient 1 iPSCs were transfected with sg4NR2E3-spCas9 and the HDR construct via lipofection. Puromycin selection was subsequently performed as described in the methods section. Following selection, cells were clonally expanded and screened for incorporation of the HDR sequence via PCR using a forward primer in the PA sequence (Figure 2A,F1) and a reverse primer outside the HDR cassette (Figure 2A,R1). Eighteen of the 25 screened clones amplified the expected PCR product, indicating incorporation of the HDR cassette (Figure S1). We chose 2 clones, clone 16 and clone 17, to confirm genomic correction. PCR was performed using primers that spanned the mutation (Figure 2A,F2,R2) and the PCR product was TA cloned. Sequencing demonstrated that all products from clone 17 still had the disease-causing c.119-2A>C mutation. However, 5 of the 12 clones from the clone 16 PCR product showed a normal sequence, indicating a monoallelic correction of the c.119-2A>C variant (Figure S2). Clone 16 was subsequently transfected with Cre recombinase to remove the floxed PuroR-vTK cassette. After a second transfection with Cre recombinase, some cells containing the PuroR-vTK cassette still remained (Figure 2C). To select for cells that lacked the PuroR-vTK cassette, cultures were treated with ganciclovir, which resulted in the removal of the PuroR-vTK containing cells (Figure 2C). PCR products using primers outside the PuroR-vTK cassette (Figure 2D,F2) and homology arm (Figure 2D,R2) demonstrated equal amounts of genomic DNA. To evaluate off-target cutting events, the top 10 off-target sites and 2 additional exonic loci selected from the top 24 off-target sites, each predicted via Benchling, were analyzed. T7E1 assays were performed with genomic DNA from unmodified control and CRISPR-corrected cells. We observed no evidence of off-target events at any of the genomic loci evaluated; that is, we observed no cleavage products that were specific to the corrected cells (Figure S3).

### 3.3. CRISPR Correction of the NR2E3 p.(Arg73Ser) Mutation in Patient-Specific iPSCs using HDR

As indicated above, the p.(Arg73Ser) mutation in Patient 2 is located 100 bps away from the c.119-2A>C mutations in Patient 1. We therefore wanted to determine if the same HDR cassette and genome editing strategy used to correct Patient 1’s iPSCs could be used to correct iPSCs generated from this individual (Figure 3A). The same plasmids were delivered; however, instead of Lipofectamine Stem, electroporation was used to determine if a different delivery method could also be used to achieve robust correction. We observed a similar efficiency with electroporation; specifically, 20 of 24 clones screened had a PCR product that indicated incorporation of the HDR cassette (Figure S4,F1,R1 primers). Patient 2 is a compound heterozygote, and only the p.(Arg73Ser) mutation was targeted using this HDR cassette. As a result, extra screening was needed to identify clones that had the HDR sequence specifically incorporated into the p.(Arg73Ser)-containing paternal allele as opposed to the maternal allele, which is wild-type at this location (Figure 3A). To identify patient-derived iPSCs that had the paternal p.(Arg73Ser) allele corrected, PCR products obtained from 6 clonally expanded iPSC lines were sequenced using the F2/R2 primer pair. Following this analysis, clone 6 was found to be corrected. None of the 18 sequencing reactions performed on DNA obtained from clone 6 contained the p.(Arg73Ser) variant (Figure S5). Not surprisingly, the p.(Arg311Gln) mutation in exon 6, which lies outside of the region of homology covered by the HDR cassette, was still present (Figure S5). A single transfection with Cre recombinase and subsequent selection with ganciclovir resulted in the removal of cells containing the PuroR-vTK cassette (Figure 3C, Cre+Gan). PCR products using both sequencing primer sets F2/R2 and F3/R3 revealed equal amounts of genomic DNA (Figure 3D,E). Using the same strategy described for Patient 1 above, Patient 2 iPSCs were evaluated for off-target cleavage events. As shown in Figure S6, indel formation was identified in OT7. However, as OT7 is located within the middle of an intronic region of a non-retinal gene, it would not be predicted to affect retinal cell development, health, and/or function. Collectively, the above-described findings indicate that a single HDR cassette can be used to successfully correct iPSCs obtained from two independent patients with separate disease-causing mutations in the gene *NR2E3*.

### 3.4. CRISPR-based Restoration of the NR2E3 Transcript in Patient-derived Retinal Cells

*NR2E3* is a nuclear transcription factor that is required for rod photoreceptor cell genesis; thus, to confirm that CRISPR-based genomic correction restores expression of *NR2E3* transcript, iPSCs (Figure 4A–C) were differentiated toward a retinal cell fate. The iPSCs generated from Patient 1, who is homozygous for the c.119-2A>C splice site mutation, were predicted to have the most overt molecular phenotype. Thus, Patient 1’s cells were chosen for the transcriptional analysis. As shown in Figure 4, at 5–6 weeks post-differentiation, three-dimensional optic vesicles generated from control (D), affected patient (E), and CRISPR-corrected (F) iPSCs appear to develop normally. To evaluate the normal kinetics of *NR2E3* expression, control iPSC-derived retinal cells were harvested at 6–20 weeks following initiation of differentiation. *NR2E3* expression is first detected after approximately 10 weeks of differentiation and is strongly expressed between weeks 13–20 (Figure 4G). After 9 weeks of differentiation, the wild-type *NR2E3* transcript was detectable in CRISPR-corrected as opposed to non-corrected iPSC-derived retinal progenitor cells (Figure 4G). By week 14, the robust expression of wild-type *NR2E3* transcript could be detected in CRISPR-corrected retinal cells (Figure 4G). Interestingly, at this timepoint, a second larger transcript could also be detected in both corrected and uncorrected cells (Figure 4G). Following gel purification and sequencing, we found that the upper band actually contained two novel transcripts that differ by only 32 bps (which explains why they appear as a single product on a 2% agarose gel). The larger transcript included 143 bps of intron 1 followed by exon 2, and the smaller contained 111 bps of intron 1 followed by exon 2. In both cases, the inclusion of this intronic sequence resulted in a frame shift and the creation of a premature stop codon just 47 bps downstream of the canonical exon 1 boundary (Figure S7). In summary, monoallelic genomic correction of the c.119-2A>C variant in patient-derived iPSCs restores the cells ability to make wild-type *NR2E3* transcript during retinal cell differentiation.

## 4. Discussion

The discovery of iPSCs, the creation of protocols for successful tissue-specific differentiation, and the development of CRISPR-based genome editing have collectively enabled scientists to study pathophysiology in human tissues like the retina that are usually inaccessible in living individuals. For example, our ability to generate photoreceptor cells from patients with molecularly-undiagnosed retinitis pigmentosa allowed us to demonstrate how a newly identified mutation in the gene male germ cell associated kinase (MAK) causes photoreceptor cell-specific disease [26]. Similarly, by generating retinal pigmented epithelial cells from a patient with suspected RPE65 associated Leber congenital amaurosis (LCA), we were able to demonstrate that a novel intronic variant in a child of Haitian ancestry, which could have easily been a non-disease causing ethnic polymorphism, altered splicing and normal transcript production [27]. In the burgeoning field of retinal gene therapy, where molecular confirmation of a patient’s disease-causing genotype is required for enrollment in a clinical gene augmentation trial, the ability to use these approaches to demonstrate that a patient truly has the disease being targeted will be invaluable.

In addition to being useful for confirming a patient’s disease-causing genotype, disease-specific phenotypes identified in patient-derived cells (which can be confirmed via CRISPR-based genomic correction) are proving to be helpful for the evaluation and development of novel therapeutics. For instance, we recently demonstrated how AAV-based replacement of *CLN3* and mitigation of the molecular phenotype in patient-derived photoreceptor cells was preferable for the demonstration of treatment efficacy to the use of an animal model that does not accurately recapitulate the disease [28]. In the current study, we demonstrate how patient-derived iPSCs and CRISPR-based genome editing can be used to evaluate the molecular phenotype associated with the rare disease, enhanced S-cone syndrome. As indicated in the introduction, enhanced S-cone syndrome is caused by mutations in the gene *NR2E3*, which disrupt rod photoreceptor cell development and result in overproduction of blue cones. By identifying how genetic alterations in *NR2E3* expression alter both disease progression and the percentage of cone and rod photoreceptor cells, we may be able to further refine our current retinal differentiation protocols.

From a photoreceptor cell replacement perspective, to restore high-acuity vision, transplantation of high-density cone photoreceptor cells will be required. There are a few promising strategies for isolating cones from retinal organoids, which depending on cell line and differentiation protocol used can have different percentages of rods vs. cones that result from using current retinal differentiation protocols (reviewed in References [29,30]); however, all of them require a large scale culture in order to obtain a sufficient number of cones following selection for retinal transplantation. A method for directing retinal differentiation along a cone selective path would be a valuable improvement. Typically, we and others have taken cues from developmental biology and designed experiments to vary the type, dosage, and timing of exogenous factor delivery as we attempt to accelerate or shift differentiation in one direction or another [31,32,33,34,35]. Although not a traditional approach, taking cues from naturally occurring genetic disease, where single base pair changes can drastically alter the phenotypic outcome, may also be useful. As we demonstrate in this manuscript, a single CRISPR-based HDR strategy can be used to correct a variety of different mutations spanning several exons of NR2E3. By comparing these changes to both in vitro phenotype and patient history, it may be possible to identify variants that selectively promote cone cell genesis with little or no associated retinal degeneration.

Although unexpected, one of the more interesting findings that we report in this study pertains to the molecular mechanism of the c.119-2A>C variant and is in opposition to a previous publication on this subject [14]. It is important to note that the previous study was performed prior to the widespread use of iPSCs and the development of CRISPR-based genome editing, and as a result, the authors elected to test the function of this variant by transiently transfecting COS7 cells (transformed green monkey kidney cells) with an expression plasmid that contained the *NR2E3* gene harboring the c.119-2A>C mutation. Transcriptional analysis in their system indicated that this mutation resulted in skipping of exon 2, which caused a frameshift and creation of a premature stop codon in exon 3 [14]. In our study, by focusing on endogenously expressed *NR2E3* in human patient-derived photoreceptor cells, we found that the c.119-2A>C mutation in fact generates an abnormal transcript that includes a segment of intron 1 followed by exon 2, which also causes a frameshift and the creation of a premature stop codon. Interestingly, analysis of the wild-type and mutant alleles using the ESEFinder 3.0 program (rulai.cshl.edu) revealed a difference in predicted splicing factor binding sites. An SRSF1 site was found to be present in the wild-type but absent in the mutant, and an SRSF6 site that is absent in wild-type was found to be present in the mutant. We suspect that the change in splice factor binding activated a cryptic splice acceptor site in intron 1 in the iPSC-derived retinal cells rather than a skipping of exon 2 as observed in the previous COS7 study. The reason that the cryptic splice site was not used in the COS7 system could simply be due to species and cell type-specific differences in the splicing machinery. That is, it is possible that the splicing machinery used by monkey kidney cells is somewhat different from that used by human photoreceptor cells. Regardless, this finding illustrates the importance of using the appropriate cell type, obtained from the species of interest, when making inferences about the function of different genetic variants. This will be especially true when attempting to determine the pathogenicity of newly identified genetic variants in the age of clinical molecular medicine.

## Figures and Tables

**Figure 1:**
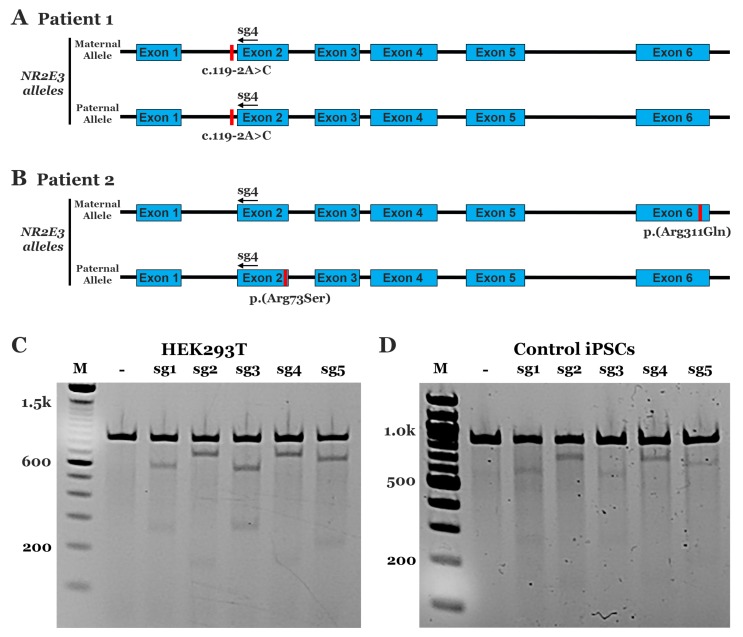
Analysis of CRISPR-Cas9 sgRNA mediated cleavage efficiency. **A,B**: Schematics depicting genomic disease-causing mutations in Patient 1—homozygous c.119-2A>C mutations (**A**) and Patient 2—compound heterozygous p.(Arg73Ser) and p.(Arg311Gln) mutations (**B**). **C,D:** Representative gel images of T7E1 assays in HEK293T (**C**) and control induced pluripotent stem cells (iPSCs) (**D**) for 5 different sgRNAs. No transfection control: (-).

**Figure 2:**
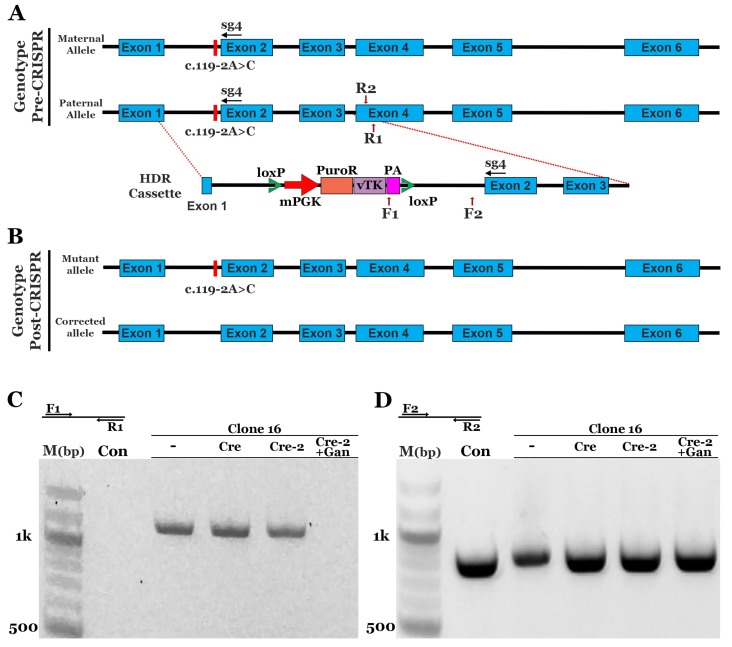
CRISPR-based homology-directed repair of the c.119-2A>C mutation in patient-derived iPSCs. (**A**): Schematic diagram depicting the genotype pre-CRISPR correction and the homology-directed repair (HDR) cassette designed to repair the c.119-2A>C mutation: Homologous sequence upstream and downstream of the loxP flanked puromycin resistance (PuroR), viral thymidine kinase (vTK), and SV40 polyadenylation (PA). (**B**): Schematic depicting the genotype following CRISPR-based repair: Monoallelic correction results in a cell line that contains one corrected allele and one mutant allele. (**C**): Representative gel image of the genomic PCR confirming incorporation of HDR cassette in clone 16 and cassette removal following transfection of Cre recombinase and selection with ganciclovir (Cre-2, +Gan). (**D**): Representative gel image of genomic PCR from the same samples presented in panel C to demonstrate similar amounts of DNA. PCR products were also used for sequencing to confirm the correction of clone 16.

**Figure 3:**
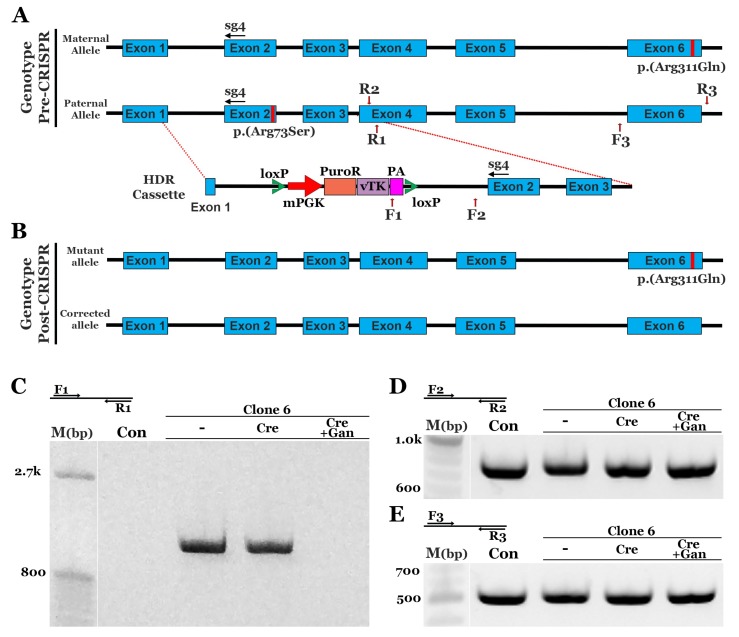
CRISPR-based homology-directed repair of the p.(Arg73Ser) mutation in patient-derived iPSCs. (**A**): Schematic diagram depicting the genotype pre-CRISPR correction and the HDR cassette used to repair the p.(Arg73Ser) mutation: Homologous sequence upstream and downstream of the loxP flanked puromycin resistance (PuroR), viral thymidine kinase (vTK), and SV40 polyadenylation (PA). (**B**): Schematic depicting the genotype following CRISPR-based repair: One corrected allele and one mutant allele harboring the p.(Arg311Gln) mutation. (**C**): Representative gel image of the genomic PCR confirming incorporation of the HDR cassette in clone 6 and cassette removal following transfection of Cre recombinase and selection with ganciclovir (Cre, +Gan). **D,E**: Representative gel images of the genomic PCR using the same samples in panel C to demonstrate similar amounts of DNA. PCR products were also used for sequencing to confirm the correction of clone 6 in one allele (**D**) and the presence of the p.(Arg311Gln) mutation in the other allele (**E**).

**Figure 4:**
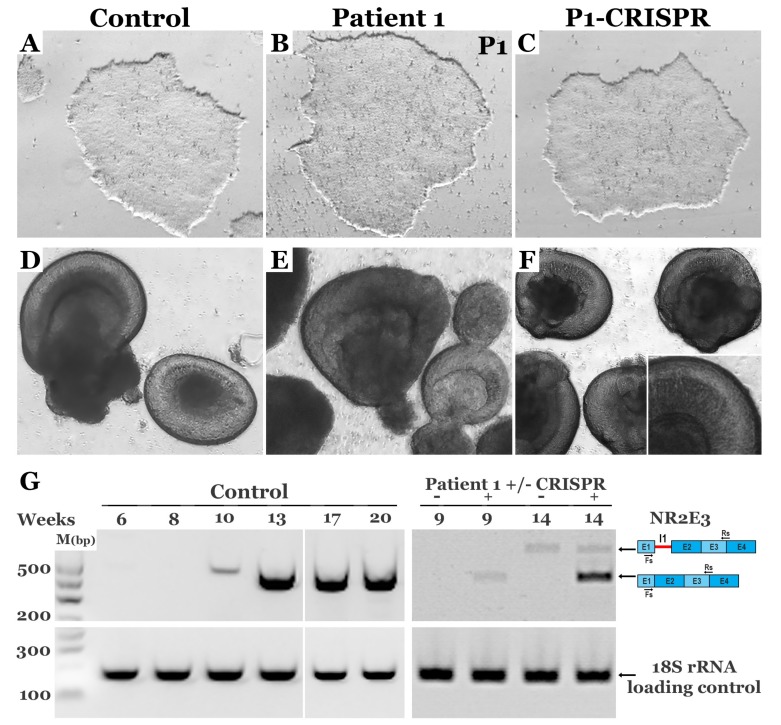
Correction of c.119-2A>C restores expression of normal *NR2E3* transcript in patient-derived retinal cells. **A–C**: Bright-field images of control (**A**), Patient 1 (**B**) and CRISPR-corrected Patient 1 (**C**) iPSCs. **D–F**: Bright-field images of control (**D**), Patient 1 (**E**) and CRISPR-corrected Patient 1 (**F**) optic vesicles at 5 weeks following initiation of differentiation. **G**: *NR2E3* transcript analysis by semi-quantitative PCR in control and Patient 1 iPSC derived retinal cells before (-) and after (+) CRISPR correction at various time points following the initiation of differentiation; 18S rRNA was amplified as a loading control.

## Supplementary Materials

The following are available online at https://www.mdpi.com/2073-4425/10/4/278/s1, Figure S1: Identification of HDR cassette incorporation in Patient 1 iPSCs., Figure S2: Sequencing of Patient 1 iPSCs., Figure S3: Off-target analysis of sg4 in Patient 1 iPSCs., Figure S4: Identification of HDR cassette incorporation in Patient 2 iPSCs., Figure S5: Sequencing of Patient 2 iPSCs., Figure S6: Off-target analysis of sg4 in Patient 2 iPSCs., Figure S7: Analysis of *NR2E3* transcript expression.; Table S1. List of sgRNAs and primers used., Table S2. List of off-target sequences, PAM sites, off-target scores, chromosomal location of off-targets and primer sequences.
