# Supplementary material for: Correction of NR2E3 Associated Enhanced S-cone Syndrome Patient-specific iPSCs using CRISPR-Cas9
Source: Genes (Basel). 2019 Apr 5;10(4):278. doi: 10.3390/genes10040278 (PMC6523438; doi:10.3390/genes10040278)
Supplement: Supplementary file 1 [file genes-10-00278-s001.pdf]

Supplemental Figures and Tables

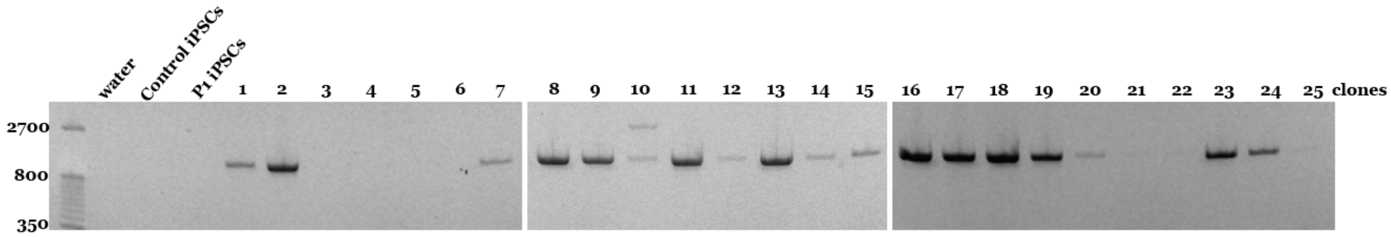

Figure S1. Identification of HDR cassette incorporation in patient 1 iPSCs. Representative gel image of genomic PCR obtained from 25 individual clones following puromycin selection. To demonstrate HDR sequence incorporation the forward primer was placed inside HDR cassette and the reverse primer was placed outside the HDR cassette (i.e. beyond the homology arm).

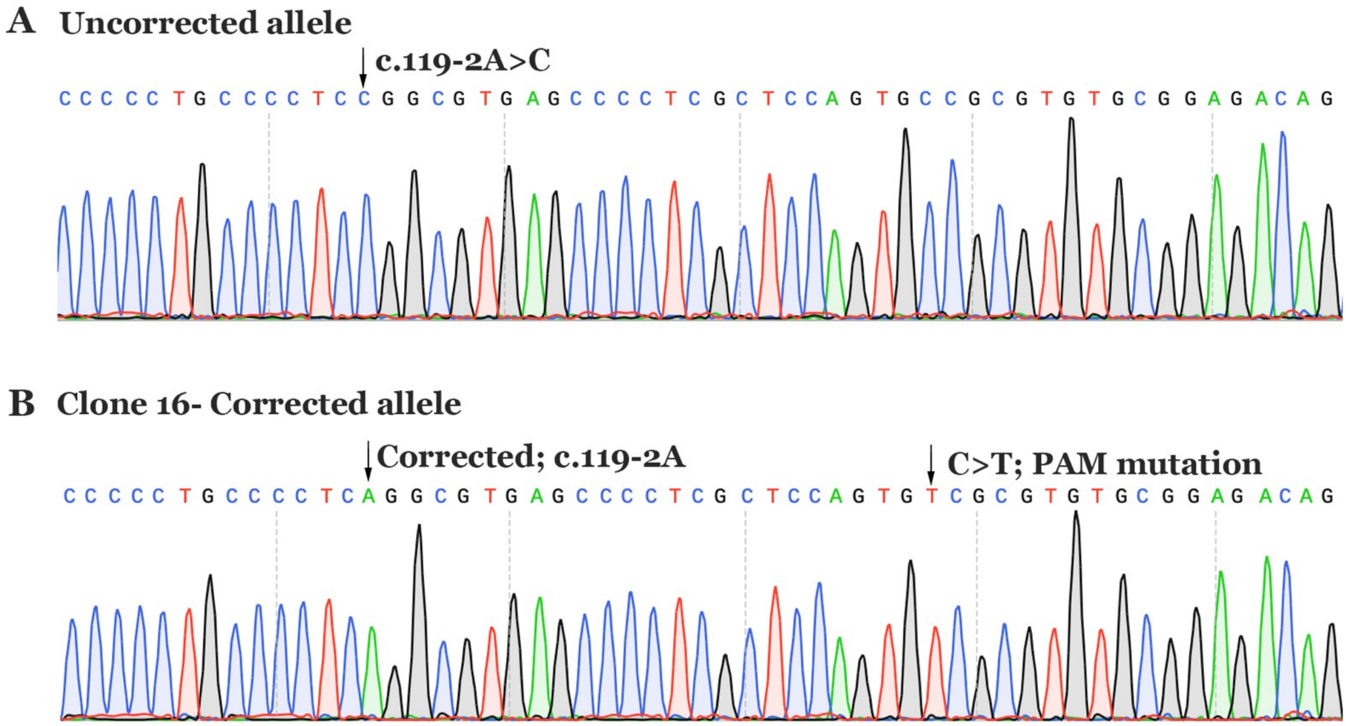

Figure S2. Sequencing of patient 1 iPSCs. Sanger sequencing chromatograms of iPSC clone 16 depicting the uncorrected c.119-2A>C containing allele (**A**) and the HDR corrected allele (**B**). Arrows indicate the location of the disease causing c.119-2A>C mutation and the synonymous C>T PAM site variation, which was introduced in the HDR cassette to prevent re-cutting events.

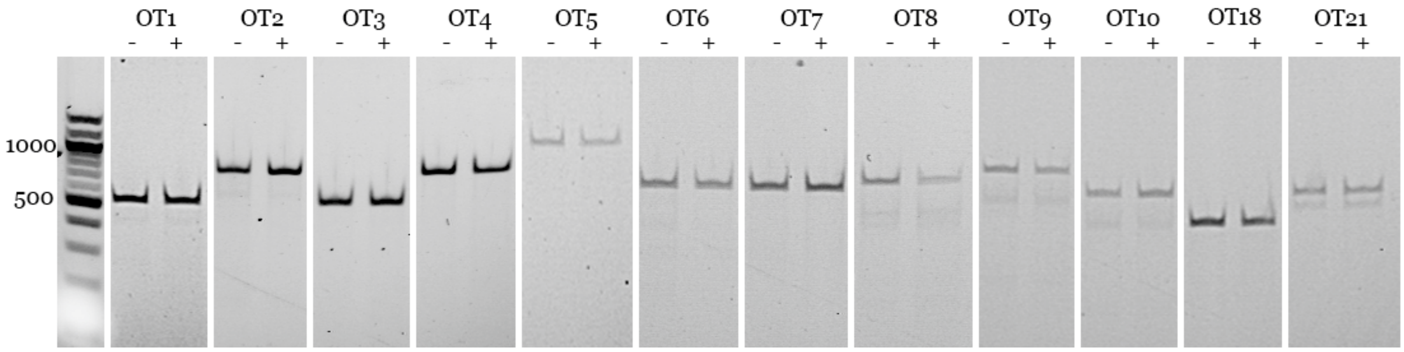

Figure S3. Off-target analysis of sg4 in patient 1 iPSCs. Representative gel images of T7E1 assays for each off-target locus in non-CRISPR treated (-) and CRISPR corrected (+) iPSCs (clone 16).

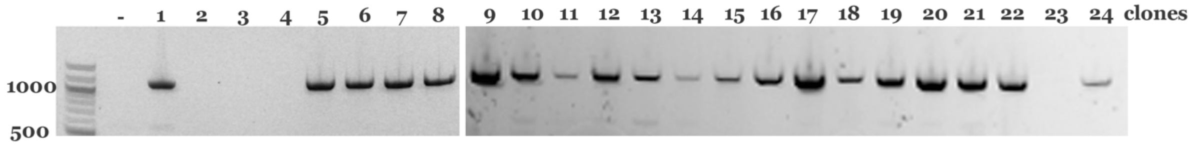

Figure S4. Identification of HDR cassette incorporation in patient 2 iPSCs. Representative gel image of genomic PCR obtained from 24 individual clones following puromycin selection. To demonstrate HDR sequence incorporation the forward primer was placed inside HDR cassette and the reverse primer was placed outside the HDR cassette (i.e. beyond the homology arm).

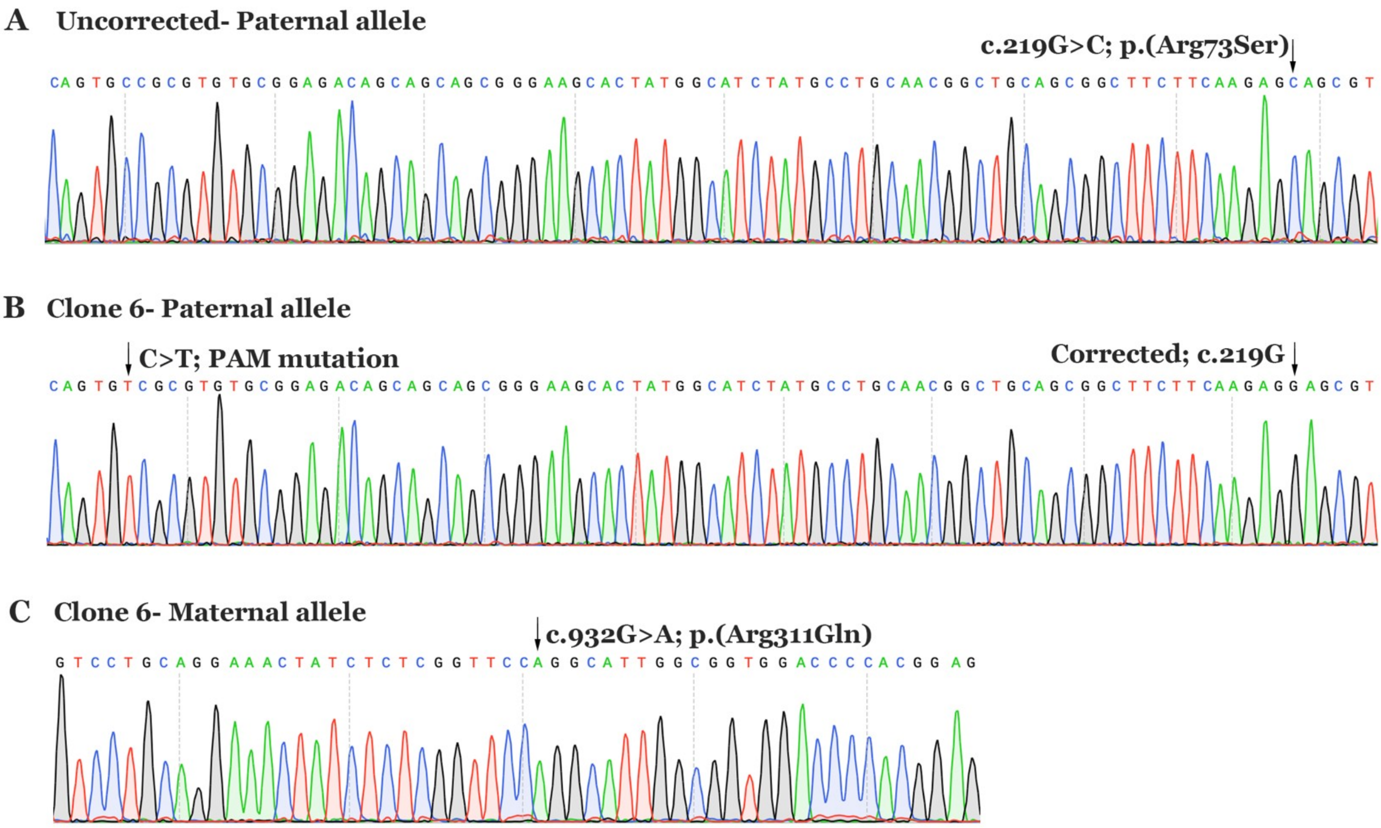

Figure S5. Sequencing of patient 2 iPSCs. A-C: Chromatograms depicting the uncorrected paternal allele (i.e., non-targeted control) containing the p.(Arg73Ser) mutation (**A**), the CRISPR corrected paternal allele of clone 6 containing both the corrected p.(Arg73Ser) mutation and the synonymous C>T PAM site variation introduced to prevent re-cutting events (**B**), and the maternal allele from clone 6 displaying the untargeted p.(Arg311Gln) mutation (**C**).

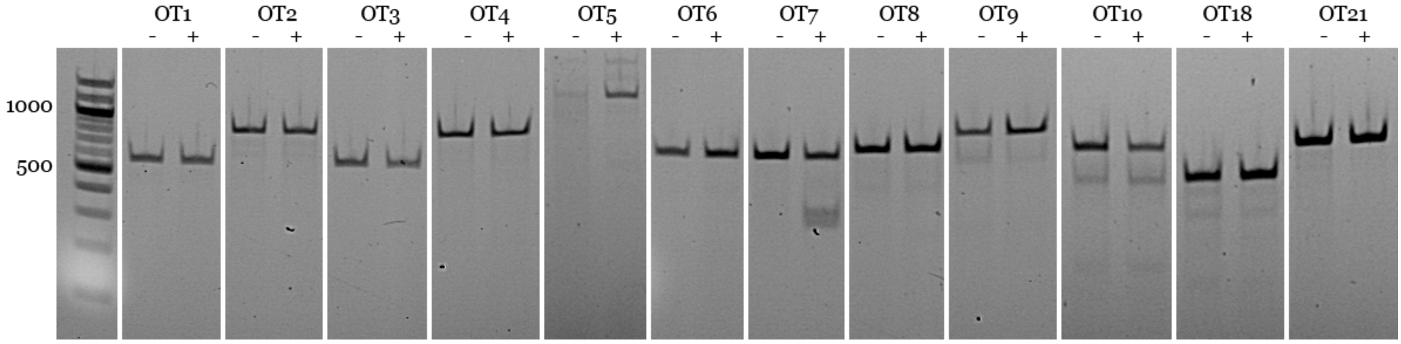

Figure S6. Off-target analysis of sg4 in Patient 2 iPSCs. Representative gel images of T7E1 assays for each off-target locus in non-CRISPR treated (-) and CRISPR corrected (+) iPSCs (clone 6).

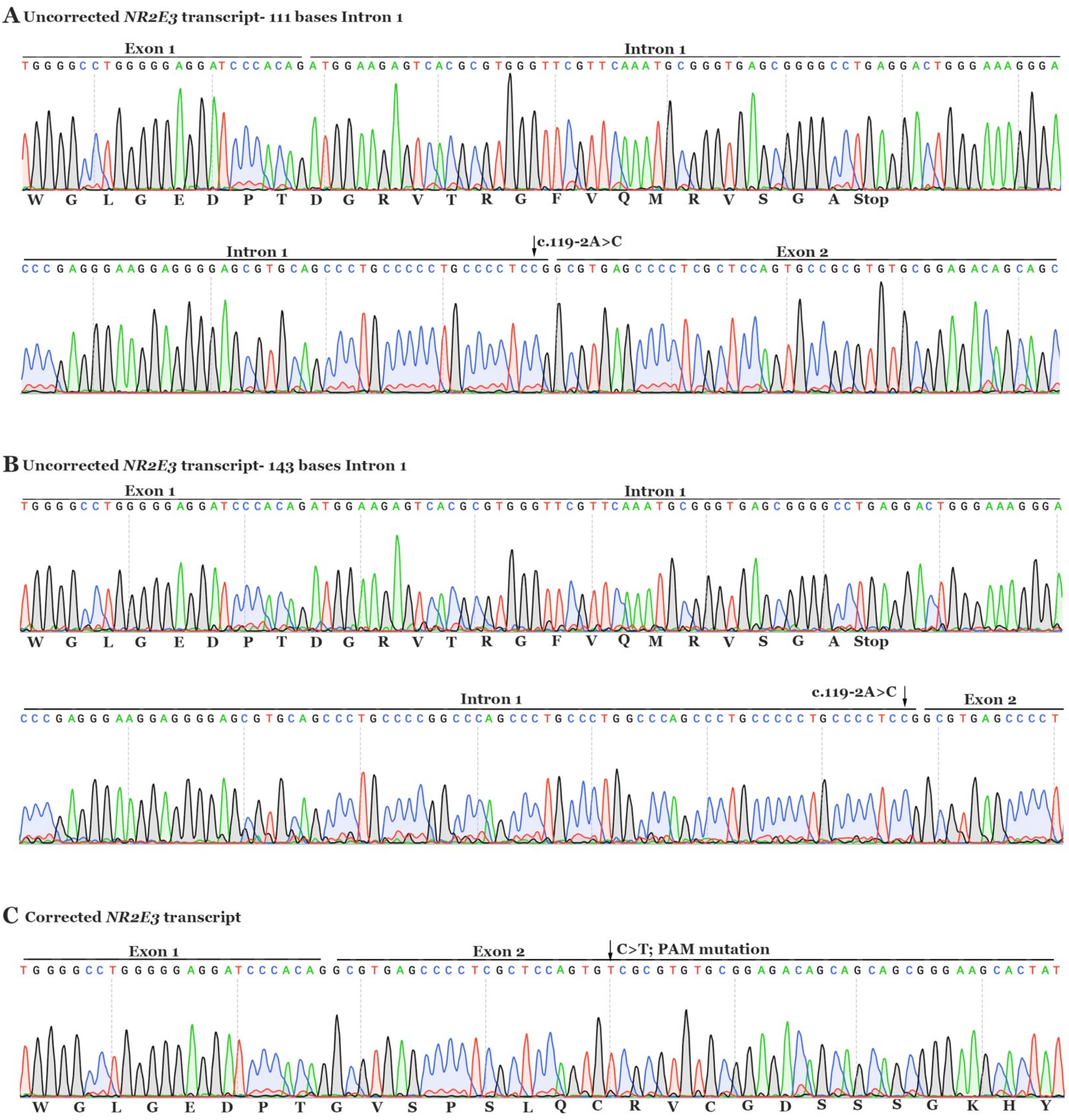

Figure S7. Analysis of *NR2E3* transcript expression. A-C: Chromatograms depicting *NR2E3* transcript sequence of uncorrected cells with inclusion of 111 bases of intron 1 (**A**), uncorrected cells with inclusion of 143 bases of intron 1 (**B**) and corrected cells demonstrating CRISPR based restoration of normal splicing in iPSC derived photoreceptor precursor cells (clone 16). Note inclusion of the synonymous C>T PAM site variation in the HDR corrected allele, which was included to prevent re-cutting events.

Purpose	Oligo name	Sequence
CRISPR guides	sg1	GACGGCGGAGGCTCATCTAC
	sg2	GGTCTCCGCACACGCGGCAC
	sg3	GCATCTACAGGTGAGTGCGG
	sg4	GGCTGCTGTCTCCGCACACG
	sg5	GATGGCATCTATGCCTGCAA
T7E1 assay	F2	GCGTGGGTTCGTTCAAATG
	R2	TCCAGCTTAGCACAGGTTTC
HDR incorporation	F1	AACTTGTTTATTGCAGCTTATAATGG
	R1	GTTATAAGGCTGGCCATGAAGTG
c.119-2/Arg73 Sequencing	F2	GCGTGGGTTCGTTCAAATG
	R2	TCCAGCTTAGCACAGGTTTC
Arg311 Sequencing	F3	GAACCCAGTGTCTCAGATGATAG
	R3	AGGATAGAGGCCTACACACA
NR2E3 RT-PCR	F4	GCACAGAGAGACAGAGGTTCAT
	R4	GTTCTGCACGGCGTCCT
18s rRNA	18s F	CGGCTACCACATCCAAGGAAG
	18s R	GCTGGAATTACCGCGGCTGCT
Sequencing	M13(-20)	TGTAAAACGACGGCCAGT
	M13R	CAGGAAACAGCTATGACC

Table S1. List of sgRNAs and primers used.

OT	Sequence	PAM	Score	Chr	F primer	R primer
sg4	TGCTGCTGTCTCCGCACACG	CGG	100	chr15		
1	TCCCGCTGTCTACGCACACG	GAG	1.52	chr19	TGGGATGCTCAGCCATTT	CCTCCTGGGTTAAAGTGATTCT
2	TGGTGCTGTGTCTGCACACG	GGG	1.09	chr12	CAGCTAGAGAGGTACAGGTAGAG	GATGAGGAAACTGAGGCATAGAG
3	TACTCCACTCTCCGCACACG	CGG	0.99	chr8	TGTCGCCATTCCCACTTC	TTAACACAGAGGGCCAAGAC
4	TGCTCCTGCCTCCGCACACC	AGG	0.98	chr17	GGTCAGGTTCTTGCCTTTCT	GACACTTCTGCCACCCTATTC
5	GGCAGCTGTGGCCGCACACG	TGG	0.85	chr19	AGATGCCGCAATGGTATGG	CAGAGGAGCCCAAGCTTTAAT
6	TGAGGCTGGATCCGCACACG	GAG	0.85	chr5	AGACATTTCCCTGGCCTTTC	ACACTGCGGAGGAAGAATTG
7	AAGTGTTGTCTCCGCACACG	GGG	0.84	chr2	CACAGGTCAGGGAAGTTTGT	ACCCTCAGCAAAGCAAGATAG
8	GGCTGCTTTAGCCGCACACG	GAG	0.82	chr13	CGGGAGCTTTGGGTTCTTTA	CGGAACTCCACCTTCCAAAT
9	TTCAGCTGTTTCAGCACACG	GAG	0.61	chr6	GAGCACTGATGGGAAGTACAA	CATCTACCACACTCAGACACAC
10	TTCTGCTGTCTCGGCACAAG	CAG	0.52	chr20	CAAGCGATTCTCCCACTTCA	CTGTAAAGCACCAAAGCACATAG
18	GGCAGCTGTCTCGGCACACA	AGG	0.38	chr1	GCAAAGGGAAGGAAGGGATTA	GTTTGTTACCCTGAAGTGGAGA
21	TGATGGAGCCTCCGCACACG	TGG	0.36	chr21	CTAGGAAGCAGGTGCAATACTC	TCAGTCCCTCCAAGACTCAA

Table S2. List of off-target sequences, PAM sites, off-target scores, chromosomal location of off-targets and primer sequences.
